# Effect of Carbohydrase Treatment on the Dietary Fibers and Bioactive Compounds of Cocoa Bean Shells (CBSs)

**DOI:** 10.3390/foods13162545

**Published:** 2024-08-15

**Authors:** Vincenzo Disca, Yassine Jaouhari, Francesca Carrà, Manuel Martoccia, Fabiano Travaglia, Monica Locatelli, Matteo Bordiga, Marco Arlorio

**Affiliations:** Department of Pharmaceutical Sciences, Università del Piemonte Orientale, Largo Donegani 2, 28100 Novara, Italy; vincenzo.disca@uniupo.it (V.D.); yassine.jaouhari@uniupo.it (Y.J.); francesca.carra@uniupo.it (F.C.); manuel.martoccia@uniupo.it (M.M.); fabiano.travaglia@uniupo.it (F.T.); monica.locatelli@uniupo.it (M.L.); marco.arlorio@uniupo.it (M.A.)

**Keywords:** dietary fiber, cocoa, enzymes, soluble dietary fiber, prebiotic

## Abstract

Cocoa bean shells (CBSs) are a byproduct of the chocolate production process, representing the external layer of the cocoa bean. CBSs exhibit many interesting chemical and nutritional characteristics resulting in a very rich content of dietary fiber (DF) and antioxidant compounds such as phenolic acids and flavan-3-ols. The DF fraction of CBSs is notably rich in soluble dietary fibers (SDFs), which may be associated with fermentability and prebiotic properties. The objective of this study was the valorization of CBSs through enzymatic treatments, thereby increasing the solubility of DF and potentially augmenting fermentability. CBSs were treated both raw and defatted. Three sets of carbohydrases were used in order to impact the dietary fiber profile. Cellulase, xylanase, pectinase and their combinations were used to perform enzymatic treatments. The application of cellulase, xylanase and a combination of both enzymes proved effective in achieving a high SDF destructuring of the insoluble dietary fiber (IDF) fraction in both defatted and raw CBSs. Notably, the SDF/IDF ratio was significantly elevated in the enzymatically hydrolyzed samples (1.13–1.33) compared to the untreated CBSs (0.33). Furthermore, the various treatments did not affect the antioxidant activity or the content of the main bioactive compounds. These results provide a foundation for new opportunities in the biovalorization of CBSs through green techniques for a range of potential industrial applications in the food and nutraceutical sectors.

## 1. Introduction

Cocoa bean shells (CBSs), which constitute 10–17% of the bean’s total weight, are derived from the removal of the external layer of cocoa beans during the industrial process called winnowing [1]. This byproduct, despite being considered as a waste material, possesses many positive nutritional characteristics. In fact, CBSs are significantly rich in dietary fibers (DFs) and proteins, with interesting amounts of several antioxidant bioactive compounds [2,3]. Even if CBSs represent a part of the cocoa plant that is not suitable for direct consumption (principally depending on the microbiological profile and the potential presence of xenobiotics and mycotoxins) and, in addition, are considered a waste material from chocolate production, the chemical composition indicates a very good set of macro/microfibers and antioxidant qualities that deserve to be considered. From a future perspective, the implementation of the use of this matrix could be crucial for the development of new CBS-based products which could claim healthy and functional characteristics. In fact, an adequate amount of DF in the human diet has been indicated as a key point in maintaining the good health of our gastrointestinal system, gut microbiota colonization and absorption of nutrients and lowers the odds of developing diseases [4,5].

The main DFs present in CBSs are pectin, cellulose and hemicellulose. As previously enlightened, fibers have been linked with many health benefits for humans, such as the improvement of gastrointestinal mobility [4], a positive influence on the microbiota regulation of the human gut [5,6]. DFs, soluble especially, are the most recognized regulators of the gut microbiota; indeed, the intestinal microbial ecosystem uses the oligosaccharides we cannot digest to produce short-chain fatty acids (SCFAs) that can actively and positively influence human health [7]. Few attempts in the literature have been reported to firstly evaluate the prebiotic potential of CBSs and secondly enhance their fermentability by the gut microbiota. The work of Disca et al. (2024) highlighted that CBSs enzymatically hydrolyzed improved SCFA production (mainly acetate and butyrate) in a simulated colon environment despite no change in the solubility of the dietary fibers [8]. Instead, the work of Younes and Karboune (2023) highlighted the possibility, through enzymatic treatment, to enhance the oligosaccharides in CBSs [9].

Besides DF, CBSs are prominently rich in bioactive compounds, which apart from theobromine and caffeine, the more representative are polyphenols (epicatechin, gallic acid and caffeic acid) known for their potent radical scavenging properties [10]. In general, enzymatic treatment is reported to be a potential strategy to augment the extraction and bioaccessibility of these compounds [11,12]. But, despite these findings, it is also reported that high and prolonged heating can affect the concentration of polyphenols and antioxidant activity [11,13].

With this background, the primary objective of the study was to investigate the effects of enzymatic treatments using three different enzymes (cellulase, xylanase and pectinase), both individually and in various combinations, on raw and defatted cocoa bean shells. The focus was to assess whether these treatments could increase the solubility of the dietary fiber fraction without compromising the antioxidant properties of the cocoa bean shells. The enzymatic treatment was aimed at enhancing the health and functional properties inherent in the substrate. This approach is in line with the principles of circular economy [14] and aims to transform cocoa shells from a mere byproduct of the chocolate industry into a novel ingredient with improved nutritional and functional properties. In essence, this research aimed to demonstrate that enzymatic treatments can effectively modify cocoa bean shells to make them more suitable for food-related industrial applications, thereby contributing to waste reduction and sustainability in the chocolate industry.

## 2. Materials and Methods

### 2.1. Materials

Dichloromethane was acquired from Carlo Erba (Rodano, Milano, Italy). Ethanol, methanol (HPLC grade), formic acid (LC–MS grade), Folin–Ciocalteu reagent, 2,2-diphenyl-1-picrylhydrazyl radical (DPPH^•^), 4-(dimethylamino)-cinnamaldehyde (DMAC), aluminum chloride (anhydrous sublimed, ≥99.8%), sodium nitrate (ACS reagent, ≥99%), Trizma^®^ base (≥99.0%), catechin (≥99.8%), epicatechin (≥99.0%), caffeine (≥99.0%), theobromine (≥99.0%), gallic acid (≥99.0%) and caffeic acid (≥99.0%) were purchased from Sigma-Aldrich (St. Louis, MO, USA). Water utilized in the procedures was obtained through a Milli-Q instrument (Millipore Corp., Bedford, MA, USA). The reagents and standard chemicals for assessing total phenol content and antiradical activity were also obtained from Sigma-Aldrich (Milano, Italy).

### 2.2. Samples

**Figure 1:**
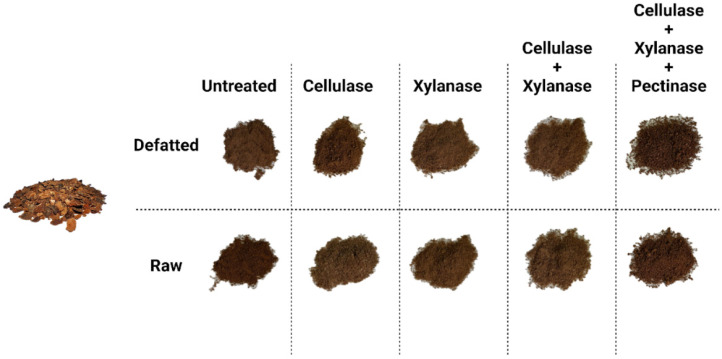
CBSs and powdered CBSs before and after the enzymatic treatments.

The CBSs were obtained as a byproduct from fermented and preroasted cocoa beans of the Nacional variety from Ecuador. They were kindly provided by an Italian leading manufacturer (Elah Dufour Novi SpA Group, Novi Ligure, Italy). Figure 1 illustrates the aspects of the shells.

### 2.3. Lipid Removal

Firstly, the cocoa bean shells were finely minced, as illustrated in Figure 1 (particle size < 250 µm) with a ball millet (MM400, Retsch Technology, Haan, Germany). The lipidic fraction removal was carried out with the semi-automatic extraction system BÜCHI B-811 LSV ( BUCHI Italia s.r.l, Cornaredo, Italy) using dichloromethane as a solvent with a cycle of 6 h.

### 2.4. Enzymatic Treatment

The enzymatic treatment was performed following the procedure of Jaouhari and colleagues [12]. Three different enzymes were selected on the basis of the structural composition of CBSs: cellulase (EC 3.2.1.4), xylanase (EC 3.2.1.8) and pectinase (EC 3.2.1.15). The enzymes were kindly donated by the Italian local supplier Tecno Food Italia (Santa Maria Della Versa, Italy). The treatments were performed in duplicate, for both raw and defatted CBSs. Four different combinations of the enzymes were tested:

- cellulase;
- xylanase;
- cellulase + xylanase;
- cellulase + xylanase + pectinase.

The enzymatic reaction was performed by equilibrating the enzymes in a buffer solution (citrate buffer pH 5; 1 mol/L) to reach the optimal conditions for enzymes activities and, for both the single enzymes and their combinations, the enzyme concentration was calculated to reach a final enzymatic activity of 16.67 µkat/L of buffer. Briefly, 15 g of CBS was hydrolyzed in 150 mL of each enzymatic solution in a thermostatically controlled shaking incubator, Shel Lab SI4 (Sheldon Manufacturing, Cornelius, NC, USA), with mild agitation (150 rpm) for 2 h at a constant temperature of 60 °C. At the end of the incubation time, the enzymatic reaction was interrupted by thermal shock (80 °C for 5 min), and subsequently the flasks were cooled on ice for 15–20 min. All the samples were stabilized by freeze drying and stored at −80 °C for analysis.

### 2.5. Total, Insoluble and Soluble Dietary Fibers

The quantification of soluble dietary fiber (SDF) and insoluble dietary fiber (IDF) was conducted using the AOAC 991.43 method with an MES-Tris buffer as described elsewhere [8]. In this procedure, the samples underwent partial digestion with alpha-amylase, followed by a subsequent digestion with protease and amyloglucosidase enzymes. Filtration was employed to remove the mass of undigested IDF, which was then dried and weighed. To precipitate the soluble dietary fiber, ethanol 95% was used. The mass of undigested SDF was isolated, dried and weighed. The determination of dietary fiber involved calculating the residue’s weight minus the protein and ash weights, expressed as a percentage of the original sample weight.

### 2.6. Extraction

Extracts of the hydrolyzed CBSs were produced as reported by Rojo-Poveda and colleagues [15]. In brief, hydroalcoholic extraction was performed utilizing a solution of ethanol (50%, *v*/*v*) in Milli-Q water. Powders were placed in 5 mL tubes in a sample/solvent ratio of 1:20 (*p*/*v*), and then were placed in a sonic bath (Branson 1515, Brookfield, CT, USA) for 30 minutes and shook for 2 h at room temperature, following a centrifugation step of 5000 rpm at 4 °C for 10 min. Finally, the filtration of the supernatant through a 0.45 µm filter was performed prior to storage of the samples at −20 °C for analysis. Extractions were performed in triplicates.

### 2.7. Total Phenolic Content

The determination of total phenolic content (TPC) followed the classical Folin–Ciocalteu assay, as outlined elsewhere [16]. Briefly, 50 µL of Folin–Ciocalteu reagent (Sigma-Aldrich) and 175 µL of aqueous Na_2_CO_3_ (5%, *w*/*v*) were added to the extracts. The solutions were then diluted with water to a final volume of 1.450 mL. The absorbance was read at 760 nm after one hour, using a Shimadzu UV-1900 272 spectrophotometer (Shimadzu, Tokyo, Japan). The results were expressed as mg catechin equivalents (CE) using a calibration curve (R^2^ = 0.9905).

### 2.8. Total Flavonoid Content

To assess total flavonoid content, the colorimetric method with aluminum chloride (AlCl_2_) was used, as reported by Rojo-Poveda and colleagues [16]. Briefly, 600 µL of opportunely diluted sample was mixed with 600 µL of NaNO_2_ (0.08 mol/L) and left to react for 5 min; then, 600 µL of AlCl3 (0.16 mol/L) was added and left to react for 6 min. Finally, 600 µL of NaOH (0.8 mol/L) was added, and the solution was well mixed. The absorbance was then read at 510 nm using a Shimadzu UV-1900 272 spectrophotometer (Shimadzu, Tokyo, Japan). The results were expressed through the interpolation of a calibration curve of (±)-catechin in mg/g of CBS dry weight (R^2^ = 0.9996).

### 2.9. Total Procyanidin Content

For the determination of proanthocyanidin (PAC) content, the protocol described by Prior et al. (2010) was used, with some modifications [17]. Phenolic extracts were opportunely diluted with a mixture of acetone, water and acetic acid (75:24.5:0.5, *v*/*v*). Then, 280 μL of diluted extract (or 280 μL of solvent for the control) was added to 840 μL of DMAC—4-(dimethylamino)-cinnamaldehyde—ethanolic solution (0.01% *w*/*v*). The solution was left to react until the maximum absorbance value was reached (20 min), at 20 °C and away from light, and then the absorbance was read at 640 nm using a Shimadzu UV-1900 272 spectrophotometer (Shimadzu, Tokyo, Japan). Results were expressed as mg of catechin equivalents (CE) through a calibration curve.

### 2.10. Antiradical Activity

The assessment of radical scavenging activity employed the DPPH^•^ assay, following the methodology reported elsewhere [15]. Briefly, 700 µL of opportunely diluted sample or methanol (control) was added to the same volume of a 100 µmol/L DPPH^•^ methanolic solution. This solution was shaken vigorously and left in the dark at room temperature for 20 min, after which the absorbance was read at 515 nm using a Shimadzu UV-1900 272 spectrophotometer (Shimadzu, Tokyo, Japan). CBS extracts were subjected to triplicate assays, and the antioxidant activity was expressed as milligrams of Trolox equivalents (TE) through a calibration curve.

### 2.11. HPLC-PDA Analysis

RP-HPLC-PDA analysis was carried out following the work of Bordiga et al., [18] with minor modification. A Thermo Scientific chromatographic system equipped with a diode array detector (Accela PDA Detector, Thermo-Fisher, San Jose, CA, USA) was used; the separation was performed on a reversed-phase Supelco Ascentis Amide column (15 cm × 2.1 mm, 3 µm, 100 Å) at 35 °C. Eluent A of the mobile phase consisted of water/formic acid (99.9:0.1, *v*/*v*), while eluent B consisted of acetonitrile/formic acid (99.9:0.1, *v*/*v*). The applied program gradient was the following: isocratic 2.5% B (10 min), from 2.5 to 12% B (10 min), isocratic 12% B (15 min), from 12% to 100% B (24 min), from 100 to 2.5% B (2 min), isocratic 2.5% B (9 min), with a total run time of 80 minutes. The flow rate and the injection volume were 400 μL/min and 5 μL, respectively. PDA detection was performed at 280 and 330, and quantification was performed based on the calibration curves obtained with the corresponding standard compounds.

### 2.12. Statistics

The statistical analysis was conducted using R software (version 4.3.0). An analysis test of one-factor variance (ANOVA) was used to determine the significant difference between samples, followed by a Tukey’s multiple comparison text. In addition, a multivariate hierarchical clustering on the principal components (HCPC) was performed.

## 3. Results and Discussion

The CBSs’ approximate composition in terms of moisture (%), ashes, fats, proteins and dietary fibers—total dietary fiber (TDF), insoluble dietary fiber (IDF) and soluble dietary fiber (SDF)—is reported in Table 1. Except for moisture content, the values are referred to in g/100 grams of CBS.

According to the study performed by Agus et al. (2018), the average moisture value in roasted cocoa bean shells is around 4.32%, a value that is very close to the moisture that has been found in the provided CBS samples of this study. A moisture below 7% has been reported to be effective in limiting microbial growth in foods, increasing storability and shelf life [19]. The average value of ashes has been reported to be 7.76 g /100 g; this value is quite similar to the 6.0 g/100 g obtained by Mellinas et al. (2020) but distant from the results of Agus et al. (2018) at almost 11% for roasted cocoa shells. Arlorio et al. (2005) have reported an ashes percentage in Italian roasted cocoa shells of around 2%, and thus very low [19,20,21].

The fat content in the provided CBSs is reported to be around 8 g/100 g; this value is again in line with that claimed for other studies, where the value is attested to be around 7% (g /100 g dry weight) [22], depending on the processing and the plants. In terms of protein content, the amount of protein in CBSs is reported to be 10%, which is slightly low compared to the various studies that have reported CBSs containing protein between 12 and 25% [21,22]. Also, in this case, the origin of the cocoa as well as the ecotype can strongly impact on this parameter. The amount of protein of a given cocoa quality may be relevant to the use of CBSs as a source of protein to be extracted and used as an ingredient in other foods [16], as an unconventional source of protein. The high value of the proteins of cocoa bean shells could be related to the absence of common allergens, considering the very low frequency of the appearance of adverse reactions to these proteins [23].

The most relevant value characterizing CBSs is represented by the fiber content. In this case, the CBS samples have a total dietary fiber (TDF) content of 58.14 g/100 g, subdivided into insoluble dietary fiber (IDF) with a value of 42.22 g/100 g and soluble dietary fiber (SDF) with a value of 17 g/100 g. The average DF reported in the literature for CBSs corresponds to the value reported for the samples in this experiment, 20–66 g/100 g dry weight of TDF, depending on the type of cocoa. The values of the insoluble and soluble fibers correspond to those reported in the literature, with 2/3 of the fibers being insoluble and 1/3 soluble [16,24]. After the enzymatic treatment performed on CBSs and defatted CBSs, some appearance differences between the samples have been registered, as shown in Figure 1.

### 3.1. Dietary Fiber Analysis

**Figure 2:**
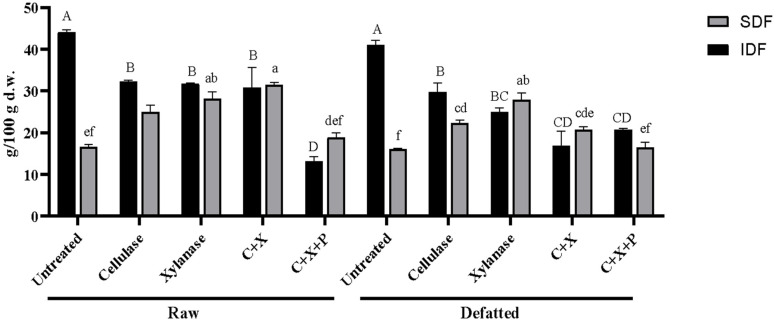
Insoluble and soluble dietary fibers expressed as g per 100 g of dry weight (mean ± SD) in enzymatically treated raw and defatted CBSs. CBSs, cocoa bean shells; IDF, insoluble dietary fiber; SDF, soluble dietary fiber; C, cellulase; X, xylanase; P, pectinase. Different letters in the same category (IDF and SDF) indicate significantly different samples (*p* < 0.05).

Figure 2 shows the measured amount of IDF and SDF in raw and defatted CBSs and the same treated with enzymes. The aim of the enzymatic treatment was to break down polysaccharides, solubilizing the DF and potentially releasing oligosaccharides that can be functionally used by the intestinal microbiota for their metabolism with many positive health effects. Most enzymes on the market catalyze the breakdown of plant cell walls to give fermentable oligo-, di- and monosaccharides depending on the concentration and treatment time; moreover, most high-performing enzymes are isolated from bacteria [25].

The results related to the IDF and SDF are reported in Figure 2, expressed as g per 100 g dw. For the raw samples, the untreated SDF content was 16.61/100 g. Treatment with cellulase increased the SDF to 24.93 g/100 g dw, and xylanase further increased it to 28.18 g/100 g dw. The combination of cellulase and xylanase (C+X) resulted in the highest SDF of 31.57 g/100 g dw, while the addition of pectinase (C+X+P) reduced it to 18.94 g/100 g.

In defatted samples, untreated SDF was 16.04 g/100 g. Cellulase treatment increased the SDF to 22.37 g/100 g dw and xylanase to 27.90 g/100 g dw. The combination of cellulase and xylanase (C+X) yielded an SDF of 20.79 g/100 g dw, and the addition of pectinase (C+X+P) resulted in 16.53 g/100 g dw. Enzymatic treatments significantly increased the SDF content, particularly with xylanase and the combination of cellulase and xylanase.

Instead, for the IDF fraction, the raw untreated CBSs showed a quantity of 44.08 g/100 g dw. Cellulase treatment reduced the IDF to 32.29 g/100 g dw, and xylanase further reduced it to 31.75 g/100 g dw. The combination of cellulase and xylanase (C+X) resulted in an IDF of 30.87 g/100 g dw, and the addition of pectinase (C+X+P) significantly reduced it to 13.17 g/100 g dw.

In defatted samples, untreated IDF was 41.01 g/100 g dw. Cellulase treatment reduced the IDF to 29.78 g/100 g dw, and xylanase reduced it further to 25.02 g/100 g dw. The combination of cellulase and xylanase (C+X) resulted in an IDF of 16.94 g/100 g dw, while the addition of pectinase (C+X+P) led to an IDF of 20.77 g/100 g dw.

Table 2 reports the SDF/IDF ratio of all the samples. This value offers insight into the proportion of soluble to insoluble fibers. Enzymatic treatments generally increased the ratio, indicating a shift toward a higher soluble fiber content. For raw samples, the untreated ratio was 0.38, which increased to 1.30 with cellulase treatment, 1.13 with xylanase and 0.98 with the combination of cellulase and xylanase (C+X). The addition of pectinase (C+X+P) reduced the ratio to 0.69.

Considering the defatted samples, the ratio in untreated samples was 0.39, which increased to 1.33 after cellulase treatment, 0.89 with xylanase and 0.81 with the combination of cellulase and xylanase (C+X). The addition of pectinase resulted in a ratio of 1.26. These changes in the SDF/IDF ratio are consistent with the literature, suggesting that enzymatic treatments enhance the solubility of dietary fibers, thus improving their functional properties and health benefits.

The combined action of the enzymes theoretically allowed the breakdown of the bonds of the main polysaccharides ofCBSs, generating smaller and soluble chains and potentially prebiotic oligosaccharides. Few works have investigated the effect of enzymatic treatments on cocoa bean shells. A study performed by Ramos et al. (2008) investigated the effect of a wide range of enzyme sets. Applying the enzymatic treatments, the researchers obtained a matrix rich in fibers which can be potentially used by human gut microorganisms to produce short-chain fatty acids. Other works have investigated the action of similar enzymes on different substrates, like in Napolitano et al. (2009) with the investigation of an enzymatic treatment effect on *Triticum durum* [26,27]. The registered increment in this matrix was almost 300% in soluble fiber fraction. A boost of prebiotic effect in postenzymatic-treated food samples has been confirmed in other works. Some examples are the experiments performed on okara (pasta obtained after soymilk squeezing, during soy milk production) where an increased prebiotic effect was registered [28] and the production of a functional strawberry-based beverage, with an increased amount of dietary fibers, mainly represented by fructo-oligosaccharides [28].

A recent work of Disca et al. (2024) highlighted that the enzymatic treatment on CBSs permitted to boost SCFA production in a simulated proximal colon environment. The production of acetate and butyrate was higher after 24 h of fermentation. Despite these findings, the enzymatic treatment did not augment the soluble fiber portion and the SDF/IDF ratio [8]. Moreover, a recent study of Younes and Karboune (2023) investigated the enzymatic generation of CBS oligosaccharides using bienzymatic and multienzymatic systems, focusing on structural characterization and prebiotic activity assessment, and enzymatically generated nondigestible oligosaccharides from CBSs showed significant prebiotic activity, promoting the growth of probiotic strains like *Lactobacillus rhamnosus GG* and *Bifidobacterium longum* [9]. In addition, a very recent study enlightened the possibility to pilot produce oligosaccharides from CBSs by isolating the pectic fraction through an alkaline treatment and hydrolyzing the fiber utilizing Depol^TM^ 670L, showing prebiotic and antioxidant properties [29].

**Figure 3:**
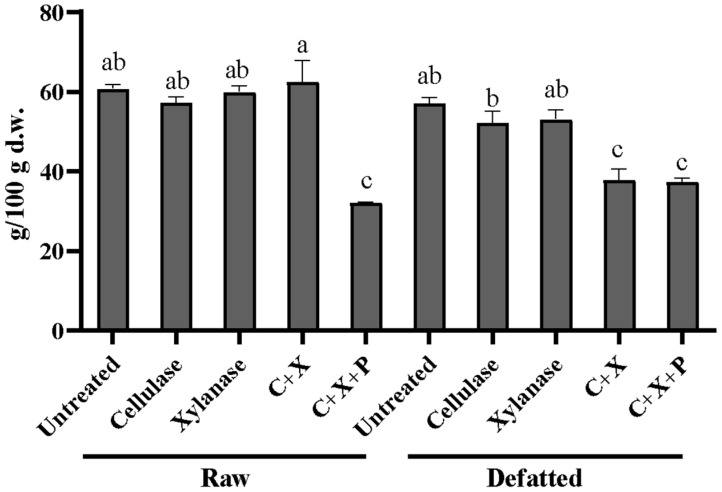
Total dietary fibers expressed as g per 100 g of dry weight (mean ± SD) in enzymatically treated raw and defatted CBSs. CBSs, cocoa bean shells; TDF, total dietary fiber; C, cellulase; X, xylanase; P, pectinase. Different letters indicate significantly different samples (*p* < 0.05).

Peculiar is the reduction in TDF for samples treated with the combination of the three enzymes (C+X+P) for both defatted and raw CBSs and the treatment with xylanase on defatted CBSs, as reported in Figure 3. This could be explained by a very strong hydrolysis that generated soluble oligosaccharides that escaped precipitation in ethanol. Similar results were obtained by Jagelaviciute et al. (2023), who treated apple pomace with a commercially available carbohydrase and obtained a decrease in IDF and SDF by obtaining oligomers and monomers that do not precipitate in ethanol [30].

### 3.2. Total Phenolic, Flavonoid, Proanthocyanidin Content and Antiradical Activity

The quantification of the total phenolic content of postenzymatic treatment CBSs and defatted CBSs has been performed through a Folin-Ciocalteu assay. The results are expressed as mg of catechin equivalents (mg/g of dry weight) in Table 3.

The results in this case were not statistically significant in terms of the difference between the untreated CBSs and the treated samples. This is a quite positive result because the amounts of bioactive phenolics in the samples are preserved despite the treatments. This indicates that the enzymatic treatment, which has been positively linked with the amounts of dietary fibers in many of these samples, did not negatively affect the total phenolic content of the samples, which remains very similar in all the cases.

The results suggest that stabilization through freeze-drying could be a solution compared to conventional oven drying. In fact, thermal stability and phenolic content has already been investigated by a study which has shown that a thermal procedure of only 60 °C has the power to decrease the total phenolic content by 15–20% [31]. Thermal treatments below or above 100 °C impact the TPC with a possible reduction of 30–50% on average [32]. Another study performed by Huynh et al. (2023) investigated the phenolic content of cocoa bean shells after a sonication procedure and enzymatic treatment with Viscozyme^®^ L (mixture of beta-glucanases, pectinases, hemicellulases and xylanases) [11]. In that case, the higher the concentration of enzymes, the more the TPC increased, while the incubation time was not correlated with a TPC incrementation. In the same study, the stabilization procedure was not investigated.

These results are very encouraging because polyphenols are very powerful antioxidants which are important in the human diet. The antioxidant power of polyphenols can decrease inflammation and prevent the development of cardiovascular and gastrointestinal diseases, as previously discussed in this work. These results can encourage future works to further investigate enzymatic treatments retaining the anti-inflammatory potential of the substrate.

The total flavonoid content has been investigated as reported in the Materials and Methods. The final concentration of flavonoids has been expressed as mg/equivalent catechin [33].

As Table 3 shows, untreated CBSs and enzymatically treated CBSs registered almost no change in the final flavonoid content. For defatted CBSs, the results are mildly different. Defatted untreated CBSs showed a greater flavonoid content than raw CBSs (9.5 and 7.6 mg/g dw, respectively), but after enzymatic hydrolysis, the defatted samples showed a similar content when compared to the raw untreated and treated CBSs, ranging from 6.5 to 7 mg/g dw. After the quantification of the total flavonoid content, it was possible to investigate how the content of flavonoids, another class of antioxidants and pigments present in cocoa and cocoa bean shells, changed after the enzymatic treatments of the samples. The results contrast with the work of Huynh et al. (2023) which registered a very low TFC in CBS samples after treatment with Viscozyme^®^ L, with a TFC around 3.5 mg/g dw that was not correlated with the enzymes used [11]. The incubation time affected more the final TFC; besides this result, increasing the incubation time did not increase the final TFC content. In another work, TFC was evaluated using sonication and modern extractive systems; the result obtained from the CBSs was 7.47 mg of rutin equivalent per gram of dry weight [34].

Antiradical activity (AA) quantification was obtained thanks to the DPPH (2.2-diphenyl-1-picrylhydrazyl) radical quenching assay. The results have been expressed as mg of Trolox equivalent (mg TE/g dw). As reported in Table 3, the results do not show any statistically significant difference between samples and treatments. These findings are very promising in terms of the preservation of the AA of samples after the treatments. In fact, as previously said, the prolonged temperature for enzymatic digestion could negatively affect this property [11]. The recent findings of Jaouhari and colleagues reported that the use of cellulase and xylanase in blueberry pomace unaffected antioxidant activity after 2 h of treatment but strongly reduced it after 4 h [12].

Proanthocyanidins were assessed as reported in the Materials and Methods and expressed as mg CE/g dw. No statistically significant variation was observed for samples enzymatically treated except for raw CBSs treated with C+X+P which showed a lower concentration for raw CBSs and a higher content for enzymatically hydrolyzed defatted CBSs versus untreated defatted CBSs.

### 3.3. Multivariate Analysis (HCPC)

**Figure 4:**
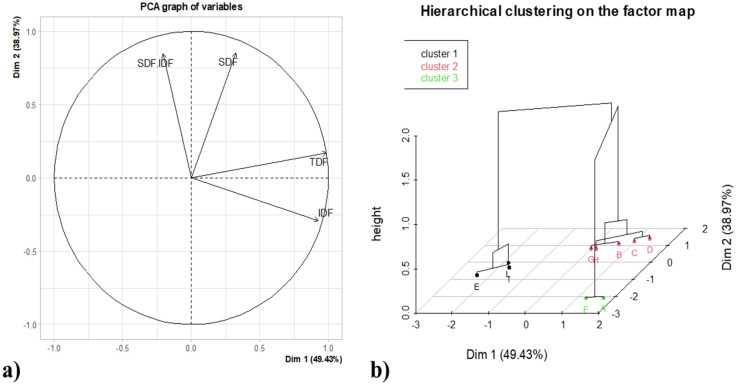
Principal Component Analysis (PCA) and Hierarchical Clustering on Principal Component (HCPC): two-dimensional loadings plot of PCA (PC1 vs. PC2) (**a**) and hierarchical clustering on the factor map obtained from corresponding PCA (**b**). IDF, Insoluble dietary fibers; SDF, soluble dietary fibers; TDF, total dietary fibers.

The multivariate analysis was carried out by applying hierarchical clustering based on principal component analysis. The statistical analysis performed was applied in order to evaluate some trends upon the various treatment samples, as reported in Figure 4. The samples have been grouped in three different clusters, depending on the IDF, SDF, TDF and SDF/IDF ratio of all samples tested; the coding of the samples is reported in the caption of Table 4.

Based on the clustering obtained (Figure 4), it is possible to appreciate that the enzymatically treated samples clustered on their own (cluster 1 and 2); on the contrary, the untreated samples clustered together (cluster 3). It is interesting to observe a cluster represented by samples that were hydrolyzed with pectinase which showed a lower total dietary fiber content. The clustering highlights the strong impact of the various treatments compared to the untreated sample more specifically, as previously discussed for the SDF portion. Finally, the removal of lipids did not appear to affect the various enzymatic treatments discussed in the previous paragraphs. The removal of the fat fraction from CBSs is a technologically important step for (i) the valorization of the residual oil for its interesting chemical properties [35] and (ii) the removal of xenobiotics and mycotoxins that may be present due to the origin, transport and storage of the ingredient [36]. Despite these observations, our results suggest that defatting may be an avoidable step for the DF valorization of CBSs.

### 3.4. RP-HPLC-PDA Analysis

Analysis through HPLC (Table 5) permitted the evaluation of selected molecules in all the samples. The molecules investigated were gallic acid, caffeic acid, epicatechin, theobromine and caffeine; the results have been expressed as µg/g dw.

The quantification was limited to the main molecules present in CBSs. No peculiar trends have been observed over the various treatments. The amount of methylxanthines in enzymatically hydrolyzed CBSs both raw and defatted seems to be conserved compared to untreated CBSs. The theobromine and caffeine levels of these samples resulted in a range with the amounts indicated in the work of Rojo-Poveda et al. (2021) on cocoa bean shells [37]. The same work highlights the epicatechin content in CBSs, which is also in the range of our obtained results. The phenolic acid concentrations seem to be in the range of the usual concentration found in CBSs as well [37].

## 4. Conclusions

The aim of this work was to investigate the effect of different enzymatic treatments on CBSs in order to evaluate the potential valorization of this food upcycled ingredient as a high-added-value byproduct. Enzymatic treatment can be a potential strategy to make CBSs available as new industrial products to add nutritional value as functional dietary fiber. The enzymatic treatments carried out in this work have indeed been shown to increase the SDF and are accompanied by a preservation of antioxidant activity.

The samples treated with cellulase, xylanase and the combination of both enzymes are those that, both for raw and defatted CBSs, showed the highest SDF yield, TDF, total phenolic content, antiradical activity and total flavonoid content. The increased quantity of SDF leaves room for further investigations, such as the full characterization of the oligosaccharide profile and the simple sugar content, largely impacting the taste as well as the fermentability properties. Furthermore, the enzymatic treatment did not negatively affect the concentrations of the main antioxidant compounds in CBSs. Further studies will be necessary to investigate the feasibility of this solution at an industrial level, but the results obtained are encouraging. The enzymatic treatments carried out could be the first step of new industrial approaches to reuse, upcycle and valorize cocoa shells, which can be exploited as new ingredients both in food and food supplements, with potential prebiotic properties that can recall the aroma and appearance of cocoa.

## Figures and Tables

**Table 1:** Raw CBSs’ approximate composition.

Parameter	Amount
Moisture (%)	4.42 ± 0.2
Ashes (g/100)	7.76 ± 0.02
Fats (g/100)	8.04 ± 0.01
Protein (g/100)	10.5 ± 0.42
Carbohydrates (g/100)	11.13 ± 0.86
TDF (g/100)	58.14 ± 1.11
IDF (g/100)	42.22 ± 0.59
SDF (g/100)	15.91 ± 0.51

**Table 2:** SDF/IDF ratio. CBSs, cocoa bean shells; IDF, insoluble dietary fiber; SDF, soluble dietary fiber; C, cellulase; X, xylanase; P, pectinase.

	SDF/IDF
Raw CBSs	
Untreated	0.38
C	1.30
X	1.13
C+X	0.98
C+X+P	0.69
Defatted CBSs	
Untreated	0.39
C	1.33
X	0.89
C+X	0.81
C+X+P	1.26

**Table 3:** Total flavonoid content, expressed as mg CE/g dw (TFC); antiradical activity, expressed as mg TE/g dw (AA); total phenolic content, expressed as mg CE/g dw (TPC); and total procyanidin content, expressed as mg CE/g dw (TPC) in raw CBSs and defatted CBSs. CBSs, cocoa bean shells; C, cellulase; X, xylanase; P, pectinase. If present, different letters indicate statistically different samples (*p* < 0.05).

	TFC	AA	TPC	PAC
Raw CBSs				
Untreated	7.69 ± 0.05 ^b^	41.34 ± 0.11 ^ab^	22.32 ± 2.49	2.33 ± 0.2 ^ab^
Cellulase	6.93 ± 0.09 ^b^	42.48 ± 3.27 ^ab^	20.37 ± 0.54	2.38 ± 0.16 ^a^
Xylanase	6.85 ± 0.09 ^b^	45.61 ± 3.28 ^a^	19.28 ± 0.17	2.37 ± 0.16 ^a^
C+X	6.58 ± 0.24 ^b^	36.1 ± 1.4 ^b^	19.52 ± 1.36	2.14 ± 0.1 ^abc^
C+X+P	6.75 ± 0.52 ^b^	42.6 ± 1.9 ^ab^	20.03 ± 0.81	1.9 ± 0.14 ^bcd^
Defatted CBSs				
Untreated	9.5 ± 0.35 ^a^	46.68 ± 0.55 ^a^	24.35 ± 1.61	1.65 ± 0.17 ^d^
Cellulase	6.75 ± 0.11 ^b^	46.51 ± 3.96 ^a^	21.3 ± 0.59	1.82 ± 0.19 ^cd^
Xylanase	7.12 ± 0.15 ^b^	42.94 ± 3.27 ^ab^	20.07 ± 2.21	2.03 ± 0.12 ^abcd^
C+X	6.8 ± 0.25 ^b^	39.41 ± 4.64 ^ab^	19.56 ± 0.47	2.01 ± 0.09 ^abcd^
C+X+P	7.15 ± 0.53 ^b^	46.67 ± 1.06 ^a^	23.3 ± 2.54	1.89 ± 0.09 ^cd^

**Table 4:** Sample coding of the HCPC analysis.

Samples		HCPC Coding
Pretreatment	Enzymatic Treatment	
Raw	Untreated	A
Raw	Cellulase	B
Raw	Xylanase	C
Raw	C+X	D
Raw	C+X+P	E
Defatted	Untreated	F
Defatted	Cellulase	G
Defatted	Xylanase	H
Defatted	C+X	I
Defatted	C+X+P	L

**Table 5:** Gallic acid, caffeic acid, Epicatechin, theobromine and caffeine quantification (expressed as µg/g dw) through HPLC-PDA. CBSs, cocoa bean shells; C, cellulase; X, xylanase; P, pectinase. If present, different letters indicate statistically different samples (*p* < 0.05).

	Gallic Acid	Caffeic Acid	Epicatechin	Theobromine	Caffeine
Raw CBSs					
Untreated	2.33 ± 0.22	1.04 ± 0.06	25.84 ± 0.3 ^a^	161.4 ± 6.31 ^ab^	29.1 ± 1.37
Cellulase	2.12 ± 0.14	1.01 ± 0.09	23.6 ± 0.41 ^b^	160.1 ± 3.22 ^ab^	25.91 ± 1
Xylanase	2.12 ± 0.18	1.02 ± 0.05	23.33 ± 0.49 ^b^	159.8 ± 4.46 ^bc^	26.13 ± 1.38
C+X	2.06 ± 0.22	0.94 ± 0.07	21.51 ± 1.36 ^b^	156.4 ± 4.24 ^bc^	25.23 ± 1.63
C+P+X	2.29 ± 0.23	0.99 ± 0.08	17.9 ± 1.14 ^c^	177.2 ± 5.7 ^a^	26.08 ± 1.97
Defatted CBSs					
Untreated	1.86 ± 0.43	0.94 ± 0.14	23.59 ± 2.45 ^b^	147.9 ± 13.9 ^c^	22.97 ± 2.55
Cellulase	1.92 ± 0.07	0.95 ± 0.11	21.96 ± 1.23 ^b^	153 ± 4.77 ^bc^	23.56 ± 0.5
Xylanase	1.96 ± 0.11	0.98 ± 0.04	23.88 ± 1 ^b^	155.3 ± 3.18 ^bc^	25.41 ± 0.32
C+X	1.93 ± 0.02	0.98 ± 0.11	22.91 ± 1.07 ^b^	156.3 ± 1.79 ^bc^	25.07 ± 0.62
C+P+X	2.19 ± 0.09	1.02 ± 0.1	21.01 ± 0.57 ^b^	156.3 ± 1.79 ^bc^	24.83 ± 0.67

## Data Availability

The original contributions presented in the study are included in the article, further inquiries can be directed to the corresponding author.
